# Supplementary material for: Characterization of the Complete Mitochondrial Genome of the Spotted Catfish Arius maculatus (Thunberg, 1792) and Its Phylogenetic Implications
Source: Genes (Basel). 2022 Nov 16;13(11):2128. doi: 10.3390/genes13112128 (PMC9690425; doi:10.3390/genes13112128)
Supplement: Supplementary file 1 [file genes-13-02128-s001.zip › genes-1963081-supplementary.pdf]

Supplementary Figure S1. Graphical illustration showing the AT- and GC-skew in the PCGs of the mtDNA of *A. maculatus*.

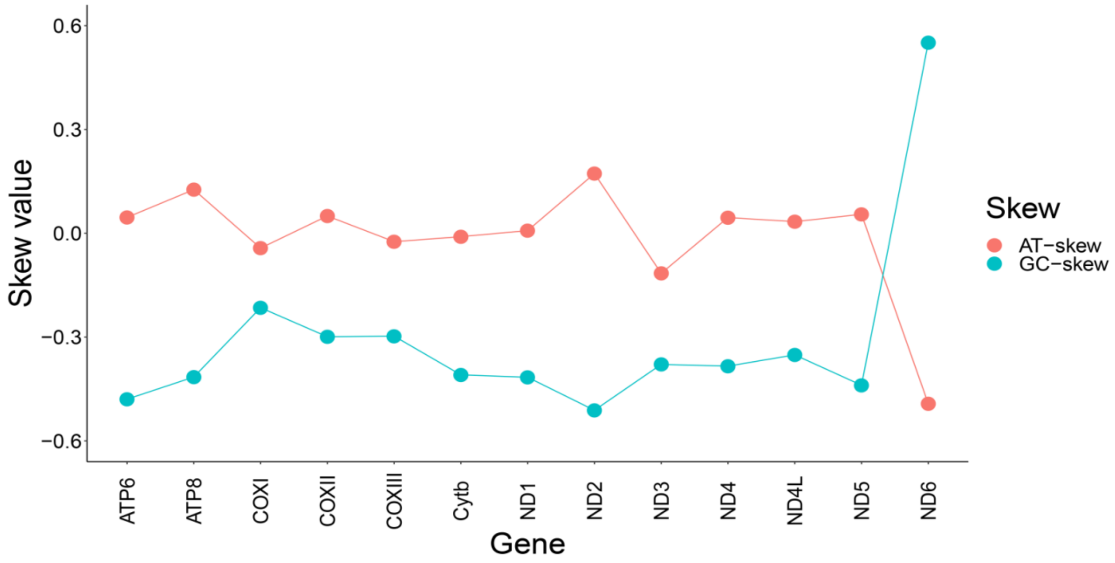

Supplementary table S1

Primers used to verify the accuracy of the assembled mtDNA sequence.

Name	Primer sequences (5′–3′)	Product length (bp)	Tm (℃)
CuF1	GAATACTACGGACGGCACTCTGAA	5104	56
CuR1	GAAAAAATAGTAAGGTCCACAGAA		
CuF2	ACCTACGACTATGCTACGCTATGA	4621	56
CuR2	AATAATCATACAACATCTACGAAA		
CuF3	GCTACCTAAACAACCGACTAATCTC	4856	52
CuR3	GAGTGGTAGTAGTTTGTGTAAGCC		
CuF4	CCTCCAAGCAGTAATCTACAACCG	5516	50
CuR4	TTTCATTTCTCTTTCAGCGTTCCC		

Supplementary Table S2 The information of Superfamily, Genera, Species, Size, Genbank number, and Identity in the Siluriformes.

Superfamily	Genera	Species	Size (bp)	Genbank No.	Identity (%)
Ariidae	Arius	*Arius maculatus*	16710	MN604079	100
		Arius arius	16711	KX211965	99.74
	Netuma	*Netuma thalassina*	16711	KU986659	99.80
	Occidentarius	*Occidentarius platypogon*	16714	NC_037469	90.14
	Ariopsis	*Ariopsis seemanni*	16830	AP012003	90.31
	Bagre	*Bagre panamensis*	16718	NC_037470	88.50
Pangasiidae	Pangasius	*Pangasius pangasius*	16476	KC572135	85.23
		Pangasius larnaudii	16471	AP012018	84.95
	Pangasianodon	*Pangasianodon gigas*	16533	AY762971	84.53
		Pangasianodon hypophthalmus	16522	KC846907	85.20
Bagridae	Horabagrus	*Horabagrus brachysoma*	16567	KU870467	83.79
		Horabagrus nigricollaris	16561	MG986722	84.35
	Tachysurus	*Tachysurus fulvidraco*	16527	HM641815	83.84
		Tachysurus intermedius	16532	KY962416	83.82
		Tachysurus ussuriensis	16536	KC188782	83.73
	Pseudobagrus	*Pseudobagrus ondon*	16534	KF514424	83.76
	Mystus	*Mystus cavasius*	16554	KU870465	83.73
	Auchenoglanis	*Auchenoglanis occidentalis*	16535	AP012005	83.85
Mochokinae	Synodontis	*Synodontis schoutedeni*	16540	AP012023	84.46
Clariidae	Clarias	*Clarias gariepinus*	16508	KT001082	84.07
		Clarias fuscus	16518	KF188424	83.68
Ictaluridae	Ictalurus	*Ictalurus furcatus*	16499	MG570460	83.40

Siluridae	Ompok	*Ompok bimaculatus*	16482	KY887474	83.40
	Silurus	*Silurus soldatovi*	16527	AB860299	83.18
		Silurus meridionalis	16526	JX087350	83.15
		Silurus asotus	16521	AP012022	83.12
Sisoridae	Glaridoglanis	*Glaridoglanis andersonii*	16532	JQ026254	83.25
